# Real-world efficacy of tirzepatide in patients with heart failure without diabetes

**DOI:** 10.1016/j.cpcardiol.2025.102998

**Published:** 2025-01-29

**Authors:** Silvio Nunes Augusto, David Kaelber, W.H. Wilson Tang

**Affiliations:** a Department of Cardiovascular and Metabolic Sciences, Lerner Research Institute, Cleveland, OH, United States; b MetroHealth System, Cleveland, OH, United States; c Center for Clinical Informatics Research and Education, The MetroHealth System and the Departments of Internal Medicine, Pediatrics, and Population and Quantitative Health Sciences, Case Western Reserve University, Cleveland, OH, United States; d Department of Cardiovascular Medicine, Cleveland Clinic, Vascular and Thoracic Institute, Cleveland, OH, United States; e Cleveland Clinic Lerner College of Medicine of Case Western Reserve University, Cleveland, OH, United States

**Keywords:** Tirzepatide, Heart failure, Prognosis

## Abstract

**Background:**

Tirzepatide, a dual agonist of glucose-dependent insulinotropic polypeptide (GIP) and glucagon-like peptide-1 (GLP-1) receptors, has shown significant cardiovascular benefits in clinical trials. This study investigates the real-world impact of tirzepatide on heart failure (HF) outcomes, leveraging data from the TriNetX platform.

**Methods:**

Using data from January 1, 2013, to December 01, 2024, we conducted a propensity-matched analysis of two cohorts of patients with HF without diabetes, where the only difference was the use of tirzepatide. The primary outcome was the incidence of acute heart failure (acute HF), with secondary outcomes including major adverse cardiovascular events (MACE), chronic kidney disease (CKD), stroke, and coronary arterial disease (CAD).

**Results:**

After propensity-matching, 897 patients were compared between the two cohorts in a 4-year follow-up, showing that untreated patients were at higher risk of incident acute HF (HR: 3.12, 95 %CI = 2.240–4.349, log-rank *p* < 0.001) and MACE (HR: 3.57, 95 %CI = 2.32–5.48, log-rank *p* < 0.001). Stroke (HR: 2.796, 95 %CI = 1.353–5.776, log-rank *p* < 0.01), CKD (HR: 1.48, 95 %CI: 1.08–2.03, log-rank *p* = 0.015), and CAD (HR: 1.474, 95 %CI,1.169–1.859, log-rank *p* = 0.001) outcomes also favored the treatment cohort.

**Conclusion:**

Tirzepatide presents a promising therapeutic option for managing heart failure, with significant metabolic and cardiovascular benefits. These real-world findings reinforce its potential role as a transformative treatment in improving clinical outcomes and quality of life for patients with HF without diabetes.

## Introduction

Tirzepatide is a novel dual agonist of the glucose-dependent insulinotropic polypeptide (GIP) and glucagon-like peptide-1 (GLP-1) receptors. It has been developed primarily for treating type 2 diabetes mellitus (T2DM) and obesity. Similar to other GLP-1 medications, evidence suggests that tirzepatide has heart-related benefits, such as reducing the risk of death and major adverse cardiovascular events (MACE).^1–5^ Recently, a meta-analysis by Taktaz et al. demonstrated that tirzepatide therapy significantly reduced the risk of MACE events. In the SUMMIT trial, tirzepatide was associated with a lower risk of cardiovascular death or worsening heart failure (HF) in patients with HF with preserved ejection fraction (HFpEF) and obesity.^6,7^ Tirzepatide has been shown to improve several cardiovascular risk biomarkers, including reductions in systolic blood pressure, C-reactive protein levels, and improvements in estimated glomerular filtration rate and urine albumin-creatinine ratio.^3^ These findings suggest that tirzepatide not only aids in glycemic control but also offers significant cardiovascular benefits, particularly in reducing the risk of heart failure and other cardiovascular events. It remains unclear if tirzepatide improves metabolic health of patients without diagnosis with diabetes, more specifically if these patients were previously diagnosed with HF. By leveraging the TriNetX platform, we sought to investigate if patients with a diagnosis of heart failure, without T2DM, may benefit from treatment with tirzepatide.

## Methods

### Data Availability.

The data, analytic methods, and study materials were collected from the TriNetX platform, which included deidentified electronic medical records from 94 healthcare organizations (HCOs) when the analysis was performed. This study utilized data from January 1, 2013, to December 01, 2024. Institutional Review Board (IRB) approval was not required as the study involved a secondary analysis of deidentified data, exempt from informed consent per the HIPAA Privacy Rule Section §164.514(a). The deidentification process was certified by a qualified expert according to Section §164.514(b)(1), with the most recent certification completed in December 2020.

#### Study design.

This observational retrospective cohort study followed the STROBE guidelines.^8^ Our analysis leveraged data from the TriNetX Research Network, a global electronic health record (EHR) platform that currently aggregates data from 106 healthcare organizations. The platform provides access to de-identified records encompassing demographics, clinical diagnoses, procedures, laboratory values, and medications.

#### Study population.

**Fig. 1.**
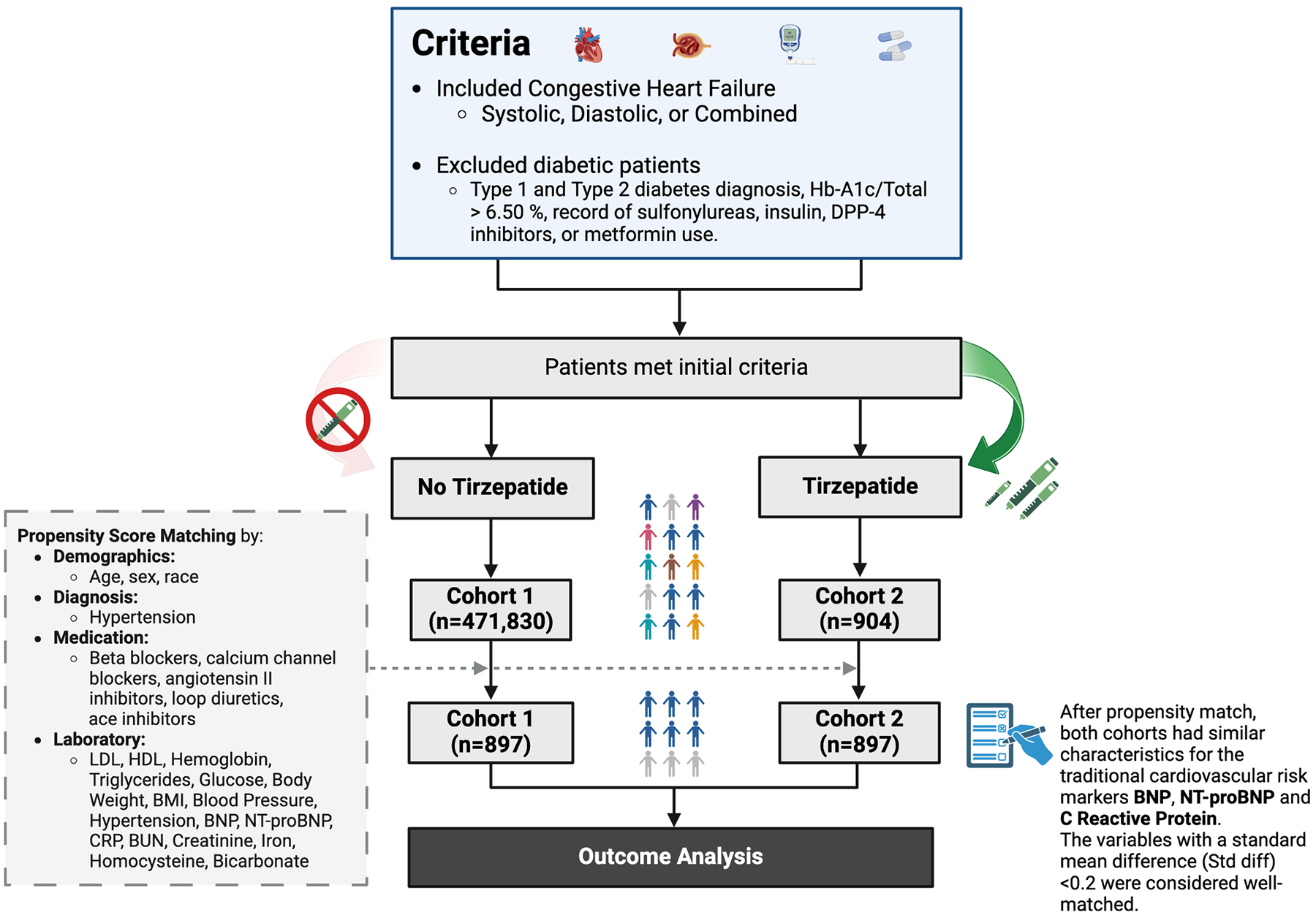
STROBE Diagram. Caption: The inclusion criteria for patients consisted of diagnosis of heart failure (systolic, diastolic, or combined). The exclusion criteria included patients diagnosed with or treated for diabetes type 1 and 2, hemoglobin A1c >6.5 %, record of taking sulfonylureas, insulin, dipeptidyl peptidase 4 (DPP-4) inhibitors, or metformin (Glucophage) medication. The only difference between cohorts 1 and 2 is that cohort 1 (*n* = 471,830) did not take tirzepatide, whereas cohort 2 (*n* = 904) consisted of patients treated with tirzepatide. After propensity score matching, both cohorts consisted of 897 patients each, and there was no significant difference between traditional cardiovascular biomarkers (BNP, NT-proBNP, and C-reactive protein).

The study included patients aged 18 to 70 years with a follow-up period of 1460 days (4 years) post-index date. The inclusion criteria included patients diagnosed with heart failure (ICD-10-CM: I.50, I50.2, I50.3, I50.4, I50.9). Patients with a prior diagnosis of diabetes, defined by one or more International Classification of Disease (ICD) encounter diagnoses of type 1 or type 2 diabetes mellitus (T2DM), Hemoglobin-A1c/Total >6.50 %, sulfonylureas, insulin, DPP-4 inhibitors, or metformin (Glucophage) use, were excluded from both cohorts. Patients in both cohorts also do not use other GLP-1 analogs during the time window. The treatment cohort included the prescription of tirzepatide, the non-treatment cohort had no prior prescriptions for tirzepatide. Fig. 1 shows the flow chart of patient selection and describes propensity score matching variables up to outcome analysis.

#### Data synthesis.

To ensure comparability between the cohorts, the most recent occurrence data was collected, and propensity score matching was performed based on the following characteristics: current age, sex, race, hypertension, medication prescriptions (beta blocker, calcium channel blocker, loop diuretics, angiotensin-converting enzyme inhibitor, angiotensin receptor inhibitor), and laboratory markers (low-density lipoprotein (LDL) cholesterol, high-density lipoprotein (HDL) cholesterol, hemoglobin, triglyceride, body weight, BMI, blood pressure, hypertension, BNP, NT-proBNP, CRP, BUN, creatinine, iron, homocysteine, and bicarbonate).

#### Study endpoints & statistical analysis.

The study outcomes included all-cause mortality, major adverse cardiovascular events (MACE) (a composite of myocardial infarction (ICD-10-CM: I21, I21.2, I21.3, I21.4), or cerebrovascular events (ICD-10-CM: I61, I63, I69, I69.3)), incident T2DM (ICD-10-CM: E11, insulin or metformin (Glucophage) medication), chronic kidney disease (CKD) progression (ICD-10-CM: N18, N18.1, N18.2, N18.3, N18.4), stroke (ICD-10-CM: I63, I67.81, I67.2), Peripheral Arterial Disease (PAD) (ICD-10-CM: I70.2, I70.20, I70.22, I70.201, I70.202, I70.29). Subgroup analysis included comparison for sex, age (cut-off: ≥ 60 years), and BMI (cut-off: ≥ 30 kg/m^2^). Risk differences, risk ratios, and odds ratios were calculated for each outcome with accompanying 95 % confidence intervals. Kaplan-Meier survival analyses were conducted to estimate survival probabilities, and hazard ratios were derived using Cox proportional hazards models. Baseline balance was assessed using standardized mean differences (Std diff), with values below 0.2 considered indicative of adequate matching. TriNetX rounds up incidence to protect patient privacy when numbers are ≤ 10, which may impact results, particularly for small cohorts and infrequent outcomes.

## Results

### Baseline characteristics.

Before propensity score matching, the non-tirzepatide cohort consisted of 471,830 patients, while the tirzepatide cohort included 904 patients. Following matching, each cohort included 897 patients, achieving balance across all baseline variables with standardized mean differences <0.2 for traditional cardiovascular biomarkers (Table 1).

### Clinical outcomes with tirzepatide use.

At the end of the four-year period, the primary outcome of acute HF was significantly better in the treatment cohort, with a survival probability of 82 % versus 80 % in the non-treatment cohort (HR: 3.12, 95 %CI: 2.24–4.35, log-rank *p* < 0.001). Patients in the non-treatment cohort were at a higher risk of major adverse cardiac events (HR: 3.57, 95 %CI: 2.32–5.48, log-rank *p* < 0.001), further supporting the protective effect of tirzepatide exposure. Chronic kidney disease (CKD) (HR: 1.48, 95 %CI: 1.08–2.03, log-rank *p* = 0.015), stroke (HR: 2.80, 95 %CI: 1.35–5.78, log-rank *p* < 0.01), and CAD (HR: 1.47, 95 %CI: 1.17–1.86, log-rank *p* = 0.001) outcomes also favored the treatment cohort.

**Fig. 2.**
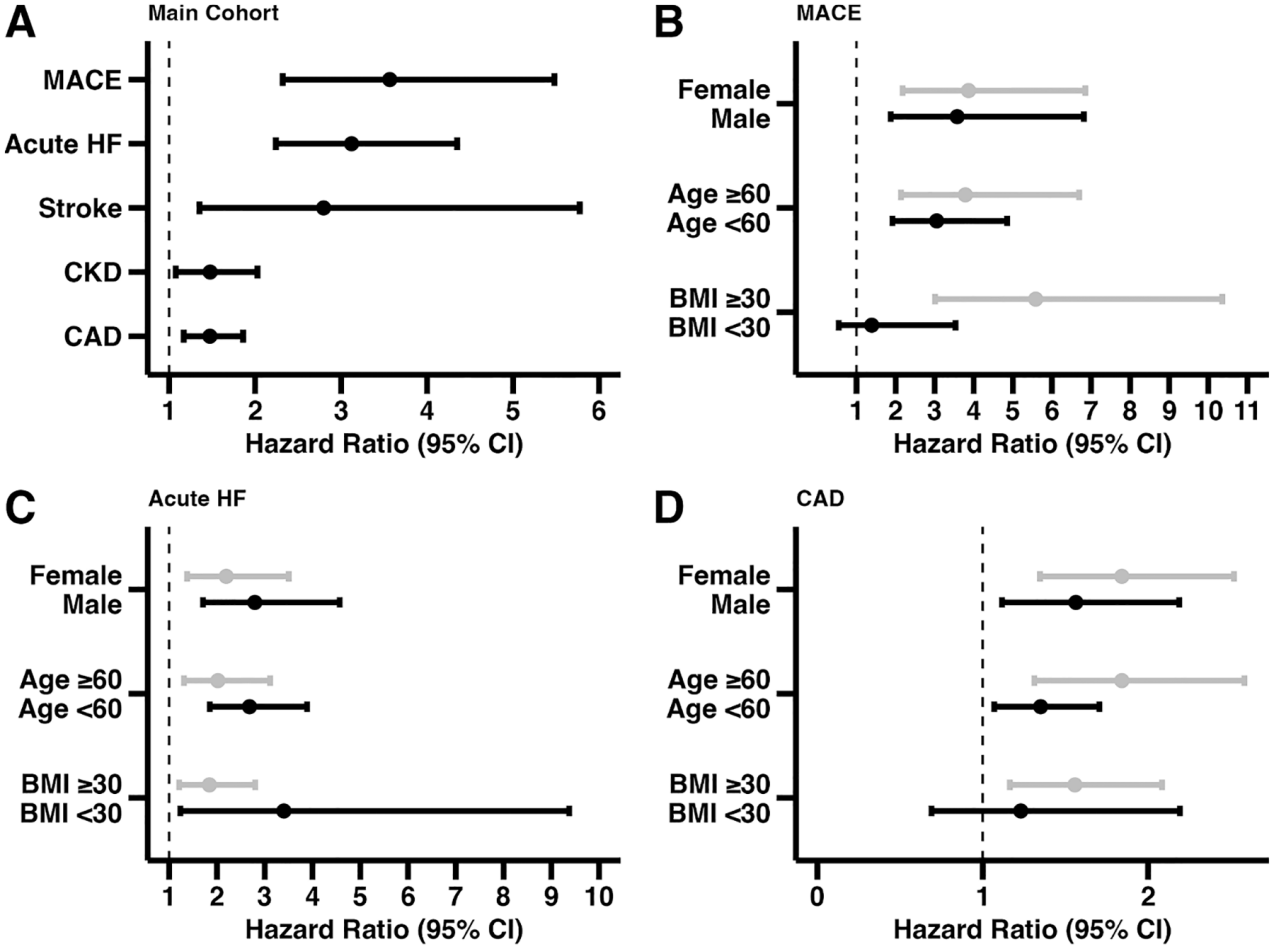
Forest Plot Hazard-Ratio for subgroup cohort analysis. Caption: Results underscore that untreated patients are at a significantly higher risk of suffering from all outcomes tested. Fig. 2A shows all outcomes of the main cohort analysis results, which includes acute heart failure (HF), major adverse cardiovascular events (MACE), stroke, chronic kidney disease (CKD), and coronary artery disease (CAD). Fig. 2B shows the subgroup analysis for MACE. Fig. 2C shows the subgroup analysis for acute HF. Fig. 2D shows the subgroup analysis for CAD. From Fig. 2B through Fig. 2D, gray bars for each subgroup indicate (1) female sex, (2) age ≥ 60 years, (3) BMI ≥ 30 kg/m^2^, Black bars for each subgroup indicate (1) male sex, (2) age < 60 years, (3) BMI < 30 kg/m^2^.

Hazard Ratios, risk differences, risk-ratios, and odds-ratios for the main cohort analysis are available in Table 2. Fig. 2A shows the main outcome hazard ratios with 95 %CI, and Fig. 2B–D show the subgroup analysis, underscoring that patients without tirzepatide are at a significantly higher risk for adverse outcomes.

## Discussion

In this analysis, we showed the safety and benefits of tirzepatide in patients with heart failure using a real-world cohort. Our key findings underscore a reduction in acute HF, risk of MACE, stroke, and CKD. Consistent with emerging evidence from recent clinical trials and mechanistic studies, the present study provides robust evidence that tirzepatide is a promising therapeutic option for patients with heart failure.

### Comparison with Recent Discoveries.

Multiple trials recently investigated the benefits of GIP/GLP-1 medications in mitigating acute and chronic cardiovascular stressors.^6,9,10^ For instance, both the LEADER trial with liraglutide and the SUSTAIN-6 trial with semaglutide showed significant reductions in MACE in patients with T2DM and high cardiovascular risk. The mechanisms underlying these benefits are thought to include improvements in glycemic control, weight loss, reductions in blood pressure, and anti-inflammatory effects.^11,12^ In the SUMMIT trial, which demonstrated tirzepatide’s ability to mitigate circulatory overload, systemic inflammation, and myocardial injury in patients with heart failure with preserved ejection fraction (HFpEF) and obesity, the survival probability remained substantially improved in the tirzepatide cohort, at the 2-year follow-up.^3^ These findings are consistent with two recent meta-analyses indicating that tirzepatide significantly reduces cardiovascular events across various patient populations, and even outperforms long-acting insulin in lowering HbA1c, fasting serum glucose, and body weight, while also reducing the risk of hypoglycemia.^6,13^

In the SURMOUNT-1 trial, a post hoc analysis demonstrated that tirzepatide significantly reduced the 10-year predicted risk of developing T2DM compared to placebo. The reductions in risk scores were observed across different doses of tirzepatide and were consistent regardless of baseline glycemic status or BMI.^10^ Jastreboff et al. results also support the dual benefits of tirzepatide for obesity and T2DM based on the significant reduction in T2DM incidence, emphasizing tirzepatide’s preventive potential.^14^ The SURPASS-CVOT trial compared the cardiovascular safety and efficacy of tirzepatide against dulaglutide in patients with T2DM and atherosclerotic cardiovascular disease (ASCVD), a population traditionally at a very high risk. Although specific stroke results from SURPASS-CVOT are not yet available, our study shows that untreated patients are at higher risk of stroke incidence, supporting the broader claim of tirzepatide’s vascular protective effects, which could also be expected in ASCVD populations.^4^ Moreover, in the SURPASS-2 trial, tirzepatide was superior to semaglutide, a selective GLP-1 receptor agonist, in reducing HbA1c and body weight.^15^

Collectively, these findings support the hypothesis that tirzepatide not only aids in weight loss and diabetes prevention but also provides critical cardiovascular protection, as reflected in the reduced rates of acute HF and MACE. This assumption is backed by mechanistic findings from *in vitro* studies on cardiac cell lines, which further supports tirzepatide’s ability to reduce cardiomyocyte injury and fibrosis under high-glucose conditions.^6^

#### Clinical Implications.

The findings from this study also reinforce the potential of tirzepatide as a transformative therapy in heart failure management, particularly among patients with comorbid obesity and metabolic syndrome. Beyond weight reduction, tirzepatide’s pleiotropic effects on inflammation, cardiac biomarkers, and cardiovascular outcomes position it as a valuable addition to current heart failure treatment paradigms. The significant survival benefit and MACE reduction observed in this large observational retrospective cohort analysis are likely attributable to tirzepatide’s unique dual-agonist mechanism, as evidenced in prior randomized clinical trials. Compared to other treatments, tirzepatide has shown greater efficacy in both glycemic control and weight reduction, suggesting a potential application in diabetes-related heart failure management.

In terms of safety, these dual agonist medications represent a potential alternative to sodium-glucose cotransporter-2 (SGLT2) inhibitors. SGLT2 inhibitors, like empagliflozin and canagliflozin, have also demonstrated significant cardiovascular benefits, but are associated with risks such as genital infections and euglycemic diabetic ketoacidosis, despite having a favorable safety profile overall.^16,17^ In contrast, GLP-1 receptor agonists are generally well-tolerated, with common side effects including gastrointestinal symptoms such as nausea and vomiting, while maintaining comparable efficacy in reducing MACE and cardiovascular mortality.^18,19^ Due to limited clinical data, GIP receptor agonists have a less well-defined safety profile, which warrants further investigation.^20^

#### Future Directions.

The findings of our study warrant validation in prospective randomized trials. The development of dual agonists targeting both GLP-1 and GIP receptors, such as tirzepatide, has shown promising results in terms of weight loss and glycemic control, which may translate into cardiovascular benefits. However, the precise cardiovascular effects of these dual agonists require further elucidation through ongoing and future clinical trials.^12,21^ For instance, the benefits of GIP medications are less well-defined for patients with cardiovascular disease. While preclinical studies suggest potential benefits, such as reductions in atherosclerosis and improvements in cardiac function, clinical evidence remains limited. A systematic review highlighted the need for further investigation into the cardiovascular effects of GIP receptor agonists (GIPRAs). Additionally, a prospective study indicated that higher fasting levels of GIP were associated with increased cardiovascular mortality, suggesting a complex and not fully understood role of GIP in cardiovascular health.^22,23^

#### Limitations.

Tirzepatide is a fairly recent medication, and its long-term effects remain to be seen. Regarding the sample size, the number of patients taking tirzepatide is considerably small compared to other more established GLP1 medications, limiting our potential to provide more extensive subgroup analysis. The small sample size of patients treated with dual agonists targeting both GLP-1 and GIP receptors is also a limiting factor. TriNetX reports all counts between 1 and 10 as 10 to prevent patient identification, affecting our ability to report expired patients for subgroup analysis. While this study leverages a large and diverse cohort from the TriNetX platform, these inherent limitations of retrospective analyses remain. The outcome of all-cause mortality should also be interpreted carefully, as a death event outside the hospital setting might not be appropriately accounted for in the system until social security number verification.

## Conclusion

This study provides real-world evidence that tirzepatide significantly reduces incidence of acute HF, MACE, stroke, and CKD over a four-year follow-up period. These findings are consistent with prior randomized trial data and underscore the potential of dual GIP/GLP-1 receptor agonists as transformative therapies in HF management. Further research is needed to elucidate their long-term effects, as well as potential benefits for stroke, CKD progression, and broader HF populations. Overall, tirzepatide offers a promising therapeutic option for patients with heart failure and obesity, providing both metabolic and cardiovascular benefits that can improve clinical outcomes and quality of life.

## Figures and Tables

**Table 1:** Baseline characteristics between cohorts before and after propensity matching.

	Before Propensity Matching				After Propensity Matching			
Variable	Non-Treatment (*n* = 471,830)	Treatment (*n* = 904)	p	Std diff	Non-Treatment (*n* = 897)	Treatment (*n* = 897)	p	Std diff
**Demographics**								
Current Age	57.1 ± 11.5	55.2 ± 10.6	<0.001	0.179	55.2 ± 12.1	55.2 ± 10.6	0.973	0.002
Male	57 %	41 %	<0.001	0.318	39 %	41 %	0.412	0.039
White	54 %	67 %	<0.001	0.270	69 %	67 %	0.418	0.038
Not Hispanic or Latino	60 %	73 %	<0.001	0.291	74 %	73 %	0.555	0.028
**Clinical**								
BMI (kg/m^2^)	30.8 ± 8.76	41.6 ± 9.53	<0.001	1.180	37.6 ± 9.41	41.5 ± 9.55	<0.001	0.414
Body Weight (lbs)	197 ± 61.2	258 ± 72.6	<0.001	0.909	233 ± 67.3	258 ± 72.7	<0.001	0.361
LVEF (%)	50.2 ± 15.4	56.6 ± 11.6	<0.001	0.469	56.5 ± 13	56.4 ± 11.4	0.952	0.006
**Diagnosis**								
Hypertension (%)	32 %	76 %	<0.001	0.977	76 %	76 %	0.782	0.013
Hyperlipidemia (%)	15 %	51 %	<0.001	0.830	40 %	51 %	<0.001	0.212
Atrial Fibrillation and Flutter (%)	9 %	24 %	<0.001	0.405	20 %	23 %	0.076	0.084
**Laboratory**								
LDL Cholesterol (mg/dL)	100 ± 38.8	99.7 ± 36.4	0.714	0.015	97.5 ± 35	100 ± 36.3	0.194	0.072
Triglycerides (mg/dL)	129 ± 120	136 ± 81.4	0.138	0.068	129 ± 82.6	136 ± 81.7	0.143	0.081
Glucose (mg/dL)	102 ± 25.2	101 ± 17.9	0.221	0.050	103 ± 19.1	101 ± 18	0.120	0.078
Hemoglobin (g/dL)	12.9 ± 2.36	13.6 ± 1.75	<0.001	0.297	13.2 ± 2.12	13.6 ± 1.76	<0.001	0.182
BNP (pg/mL)	504 ± 2,462	179 ± 311	0.033	0.185	791 ± 4,481	181 ±312	0.030	0.192
NT-proBNP (pg/mL)	2,281 ± 6,040	846 ± 2,837	<0.001	0.304	1,186 ± 3,060	811 ± 2,827	0.182	0.127
C-Reactive Protein (mg/L)	31.8 ± 55.9	23.1 ± 45.7	0.018	0.169	29.4 ± 58.1	23.1 ± 45.9	0.188	0.120
Bicarbonate (mmol/L)	25.8 ± 3.68	26 ± 3.5	0.132	0.055	26 ± 3.68	26 ± 3.5	0.781	0.014
Iron (μg/dL)	67.7 ± 49.4	68.7 ± 37.8	0.729	0.024	70.2 ± 57.1	68.3 ± 38	0.666	0.038
BUN (mg/dL)	17 ± 11.3	16.5 ± 7.76	0.221	0.051	17 ± 10.5	16.5 ± 7.77	0.219	0.062
Creatinine (mg/dL)	1.2 ± 1.94	0.958 ± 0.712	<0.001	0.163	1.07 ± 1.15	0.958 ± 0.714	0.018	0.119
GFR (mL/min/1.73 m^2^)	84.5 ± 28.6	83 ± 22.7	0.185	0.058	80.5 ± 25.5	83 ± 22.7	0.065	0.104
**Medications**								
Beta blockers (%)	30 %	71 %	<0.001	0.894	70 %	71 %	0.642	0.022
Loop diuretics (%)	18 %	53 %	<0.001	0.786	51 %	53 %	0.508	0.031
Angiotensin II inhibitors (%)	11 %	46 %	<0.001	0.858	44 %	46 %	0.476	0.034
ACE inhibitors (%)	17 %	36 %	<0.001	0.435	35 %	35 %	0.843	0.009
Calcium channel blockers (%)	16 %	42 %	<0.001	0.595	42 %	42 %	0.962	0.002

Numerical data is expressed in mean ± standard deviation, and categorical data as count (%). Abbreviations: Std diff, standard difference; BMI, body mass index; LDL, low-density lipoprotein; HDL, high-density lipoprotein, BNP, B-type natriuretic peptide; NT-proBNP, amino-terminal pro-B-type natriuretic peptide; GFR, glomerular filtration rate; ACE, angiotensin converting enzyme.

**Table 2:** Risk Difference, Risk Ratio and Odds Ratio for Primary (Acute HF) and Secondary Outcomes (MACE, CKD, Stroke, PAD, CAD) Comparing Non-Tirzepatide Cohort to Tirzepatide Cohort.

	Risk Difference (RD)				Risk Ratio (RR)		Odds Ratio (OR)		Kaplan-Meier Survival Probability				
Outcomes	RD	95 %CI	z	p	RR	95 %CI	OR	95 %CI	Treatment	Non-Treatment	X^2^	df	p-value
Acute HF	9.92 %	(7.16 %,12.68 %)	6.943	< 0.001	2.894	(2.104,3.979)	3.232	(2.287,4.567)	80.06 %	81.72 %	50.351	1	< 0.001
MACE	7.14 %	(4.86 %,9.40 %)	6.096	< 0.001	3.37	(2.216,5.127)	3.638	(2.343,5.649)	82.85 %	91.59 %	38.464	1	< 0.001
CKD	3.23 %	(0.61 %,5.86 %)	2.409	0.016	1.446	(1.069,1.957)	1.498	(1.076,2.086)	75.46 %	83.72 %	5.905	1	0.015
Stroke	1.90 %	(0.58 %,3.21 %)	2.824	0.005	2.7	(1.315,5.545)	2.753	(1.325,5.721)	96.06 %	97.87 %	8.415	1	0.0037
CAD	5.35 %	(1.93 %,8.77 %)	3.061	0.0022	1.39	(1.124,1.719)	1.482	(1.151,1.909)	35.86 %	65.54 %	10.879	1	0.001

Abbreviations: MACE, major adverse cardiovascular events; CKD, chronic kidney disease; CAD, Coronary artery disease; RD, Risk difference; RR, Risk ratio; OR, Odds ratio.
